# Carcinoma Lenticulare—Cancer En Cuirasse (Velpeau)

**Published:** 1891-07

**Authors:** 


### Selections.

### CARCINOMA LENTICULARE—CANCER EN CUIRASSE (VELPEAU).

On the 12th of March, 1890, Mrs. F., aged 55, of Pratt Mines, near Birmingham, Ala., came to me presenting the following history and symptoms: Duration of the disease was about two years. She had first noticed a "hard lump" in the skin of the left breast. About the same time "red spots" appeared in the skin of the left mammary and pectoral region, followed by similar condition on the right breast. In these "spots" papules and tubercles were formed and the skin became diffusely infiltrated. A gland in the left axilla became enlarged and "tender" a few weeks ago. She could not state how long other enlarged (cervical) glands had been so.

At present the skin of the entire anterior thoracic region is involved, the left side, however, more extensively than the right. The right mammary gland is decidedly indurated, the left apparently so, but the skin is so hard, dense, and adherent that the real condition of the gland is difficult of determination. The skin over the gland is quite immovable. Deeply set in this densely-infiltrated skin of the thorax are numerous tubercles and nodules, studding the surface. External to the lateral borders of infiltration are a few isolated tubercles and nodules, circumscribed by normal-looking skin. Some of these are elevated and of purplish color; one is umbilicated, others are depressed; the cutaneous covering is movable over them, and they feel like pieces of cartilage set in and occupying the subcutaneous tissue. The color of the "older" portions of the diseased surface is a purplish to dirty-gray, shading off, in a few newer, isolated lesions, to that of the normal skin. The areolæ of the nipples are infiltrated and slightly scaly, and the nipples are similarly affected, but are not retracted. In the more densely infiltrated area the subcutaneous tissue is as completely involved as the skin, but many of the solitary lesions seem confined to the skin and are freely movable. The tubercular or nodular lesions, both in the infiltrated and non-infiltrated skin, vary in size from a pin-head to that of a small cherry. Many of the larger nodules have in their summit a peculiar round, shot-like, waxy-looking formation. A few dilated vessels can be seen on the surface of some of the lesions. Just beneath the left clavicle there is a reddish, waxy-looking, very hard growth the size of the finger-tip, the summit irregular and showing one or two dilated vessels—the whole appearance suggesting epithelioma. The infiltrated portion is limited below by a line corresponding to the border of the diaphragm, and the edge is as sharply defined as that of a board. The clavicles roughly form a line of limitation above, while a line dropped from each axilla would define the lateral limits.

On the inner surface of the left arm, which is œdematous, an irregular-shaped, deep red, non-elevated patch is seen, very itchy and scaly from scratching. The patient says this is the characteristic appearance and sensation of the skin when it is first invaded by the disease. On the back are two or three similar patches, in one of which slight infiltration appears to have already occurred. Just to the left of the external border of the left scapula is a bean sized, roundish lesion, smooth and of pinkish color, surrounded by skin of normal appearance, none of the "primary redness" of skin being present. (This lesion was excised for microscopic examination, and seemed limited to the corium.) As the disease progresses in the above-mentioned red patches itching is suceeeded by pain and a sensation of heat. There is also pain in the breasts. There has not—to this time—been the slightest ulceration in the diseased tissues. Anterior cervical glands are enlarged and hard. Right axillary not involved. Patient has lost a little in weight, but has none of the look of "cancerous cachexia." The only general smyptons are constipation and "dyspepsia."

She is the mother of four children, the youngest now twentyfour years old. During the lacation with her first child the right breast became inflamed, suppurated, and was incised.

The patient returned home in two days. Naturally the prognosis was unfavorable, and the only treatment which seemed to be indicated was that directed toward the general health of the patient. Some months later I learned practically no treatment had been followed. Under date of January 5th, 1891, Dr. G. W. Brown, of Pratt Mines, wrote that ulceration took place in older portions of diseased surface soon after the patient returned home, and "the nodules increased in number and extent." Patient died December 1st, 1890.

*Microscopic Examination.*—The above mentioned specimen was hardened in alcohol and imbedded in celloidin. Sections were stained with "borax carmine" and hæmatoxylin solution, the best picture being obtained with the former. Epidermis normal. In papillary layer of corium a few issolated, scattered connective-tissue and round cells, as may be seen in slight inflammation. About hair-follicles there are collections, in groups of small cells, round, spindle, or irregular in shape. At some points in the papillary layer there are compact groups of chiefly round cells, in which no blood-vessel or capillaries can be seen. The sweat-ducts seem free. The whole system of cutaneous vessels is more or less affected. The papillary branches, the superficial and the deep plexus and their connecting branches are surrounded or completely enveloped by small round cells, among which are seen a few of the spindle variety. These cells invest the vessels in a thin layer throughout their course or expand into groups. The "groups" are especially numerous about the papillary branches. Every visible branch in the field has its cell accompaniment. A little above and also at the level of the sweat-glands are cells of a different type and larger, round, oval, or polygonal in shape, and either in "bands" or roundish groups closely confined by connective tissue. The cell nucleus is clear, and an occasional nucleolus may be seen. These bands and groups of cells, with their connective-tissue covering, are so arranged, as to form a somewhat reticular image. In many of these are to be seen, probably around a capillary, small cells of the type already mentioned. The character and arrangement of the large cells is such as we usually see in tissues just becoming infiltrated with carcinomatous disease.

The sweet-glands proper were normal, save for the presence of the small cells accompanying their nutrient vessels.

A specimen from an older portion of the disease might have been better, but I believe the one obtained gave a sufficiently clear aid to reaching the diagnosis made.

I will admit that I first believed the disease to be sarcoma, being influenced by the macroscopic as well as by the microscopic appearances (condition of the blood-vessels), and especially by the "primary" redness in areas of beginning disease. A portion of Funk's (*Monatshefte für prak. Dermatologie*, Band VIII., Nos. 1 and 2) description of cutaneous sarcomas fits this case fairly well. He says, under "Early Forms, Primary Efflorescence;" "*a*. A yellowish-red, red, brown or bluish-red, generally pea-sized macule. Many of these are produced wholly from small tortuous vessels, many are hemorrhagic. The onset of skin sarcoma may frequently be confounded with purpura. *b*. A millet-seed-sized smooth papule may arise from the macule or its border." Under "*c*" he says: "The tubercles of sarcoma are generally pea-sized, 'level' (more rarely hemispherical), hard and smooth, dark bluish-brown color. Recent tubercles frequently translucent with tortuous vessels on the surface, of bright color, lesions becoming darker gradually as result of exudation of blood. Older tubercles frequently of the blackish-violet color." Under "*e*. A diffuse infiltration of the skin," he says: "The affected part is slightly raised, of a bluish-red-brown color, hard, board-like, and not at all or only slightly flexible. Diffuse infiltration generally involves the subcutaneous connective tissue also."

Crocker, ("Diseases of the Skin," 1888) gives the symptoms of carcinoma lenticulare and the stage answering to Velpeau's cancer en cuirasse, and quotes the typical case published by Morrow and Robinson in this *Journal* in Vol. II., p. 1, 1884. This description so well covers the symptoms seen in my case that quotation of further authorities would be superfluous. My memory of Funk's description first led me to think of sarcoma, and then Crocker's induced a further study, of the symptoms ; and microscopic appearances led to the discovery of the carcinomatous nature of the disease and to giving the age of the patient, the enlarged lymphatics, and the epitheliomatous character of one of the lesions their full share in the symptomatology.—*M. B. Hutchins, M. D., Atlanta, Ga., Journal of Cutaneous and Genito-Urinary Diseases.*

COMPOUND TINCTURE OF CINCHONA IN RHEUMATIC FEVER. —C. C. P. Clark (*Therapeutic Gazette*, December 15, 1890) reports three cases of rheumatic fever treated with Huxham's tincture of cinchona, in which the happiest results were obtained after other measures had failed. One of the cases was that of a boy fourteen years of age; the other two were adults. All recovered entirely in less than a week.

The medicine was given in half-ounce doses every four hours. The author states that he has cured a dozen more patients with almost exclusively the same treatment, and further remarks that the locality of his practice is free from malaria. The effects observed in the disease referred to are attributed solely to the action of the cinchona tincture.—*Univ. Med. Magazine.*

NATIONAL MEDICAL EXAMINERS' ASSOCIATION.—This organization, begun in Washington, D. C., May 6th, has for its object, if possible, to harmonize all the laws of all the States regulating the practice of medicine, so as to adopt something of a uniform standard for all parts of the United States. Dr. J. H. Rauch, of Springfield, Ill., is President, and Dr. L. J. Picot, of North Carolina, Secretary. The other representatives present were Drs. Jerome Cochran, Alabama; C. R. Oglesby, Florida; J. C. Shroeder, Iowa; P. H. Millard, Minnesota; George Homan, Missouri; ——— Cole, Montana; W. P. Watson, New Jersey; W. W. Potter and ——— Payne, New York; and Hugh M. Taylor, Richmond, Va.—*Virginia Medical Monthly.*

ANTIPYRIN IN INFANTILE ENURESIS.—Dr. J. Bouisson (Theses de Lyon) states that the effect of antipyrin in the treatment of the enuresis nocturna of childhood is "simply marvellous." The remedy is exhibited in doses of 10 grains, repeated to the third time (30 grains in all) at intervals of one hour, commencing four hours before bedtime. Of eight inveterate cases in which the disease had existed for several years, and upon which every other remedy and method of treatment had proved futile, every case was completely cured. Several months have elapsed since the treatment, and in no case has there been a relapse, nor have any symptoms of return been noted.—*National Druggist.*

AMERICAN MEDICAL ASSOCIATION.—The session in Washington, D. C., May 5–8, was a decided success. Officers for 1891–2 are: President, Dr. H. O. Marcy, of Boston. Vice-Presidents, Drs. Willis P. King, of Missouri; Henry Palmer, of Wisconsin; W. E. B. Davis, of Birmingham, Ala.; W. E. Taylor, of San Francisco, Cal. Treasurer, Dr. Richard J. Dunglison, of Philadelphia, Pa. Secretary, Dr. Wm. B. Atkinson, of Philadelphia, Pa. Place of Meeting, 1892, etc., Detroit, Mich., first Tuesday in June, 1892. Dr. H. O. Walker, Chairman of Local Committee of Arrangements.

GONORRHŒA.—I have found nothing equal to hydrastine in treatment of gonorrhœa.

R. Hydrastine, gr. i–ij.

Zinci acetatis, }

Plumbis acetatis, } aa gr. i.

Aquæ puræ.

M. Sig.—Use as injection after washing out urethra with warm water. Internally, alkalies and copaiba.—*McIntosh, N. Y. Medical Journal.*

THE Medical Department of Tulane University, New Orleans, has been the fortunate recipient of $100,000 from Mrs. Richardson, wife of Dr. T. G. Richardson, Dean of the College. The gift will be used in erecting new buildings.

DR. HOBART A. HARE has retired from the editorship of the Philadelphia *Medical News*, and is succeeded by Dr. Geo. M. Gould.

THE Faculty of the Harvard Medical School have decided that, beginning with the class entiring in September, 1892, the regular course necessary to obtain the medical degree shall be four years.

DR. THOS. P. GARY, of Ocala, Fla., died on the 10th ult. Dr. Gary was President of the Florida Medical Association, and one of the best known and most respected gentlemen of the Florida profession.

DR. JOS. P. LOGAN, of this city, died June 2d. During his active career Dr. Logan was universally beloved, and recognized as one of the ablest of the Atlanta physicians. He passed away, full of years and honors.

DR. WM. PERRIN NICOLSON, Dean of the Southern Medical College, was united in marriage, on the 10th inst., to Miss Carolyn Clayton Crane, of this city. The wedding was described by the daily press as one of the most brilliant ever seen in the State. To the happy pair the JOURNAL extends its hearty congratulations and most cordial good wishes.

THE Medical Faculty of the University of Pennsylvania are making an effort to establish the four-year course. To that end, Provost William Pepper proposes to give $50,000 toward an endowment fund of $250,000, and $1,000 annually for five years toward a guarantee fund of $20,000. This offer is made on condition that an obligatory four-year course of study be established before September, 1893. Other members of the faculty have subscribed $10,000 toward the guarantee fund, which is intended to cover any deficit accruing out of the new arrangement.

